# Quantification of Tyrosine in Pharmaceuticals with the New Biosensor Based on Laccase-Modified Polypyrrole Polymeric Thin Film

**DOI:** 10.3390/polym14030441

**Published:** 2022-01-22

**Authors:** Ancuța Dinu, Constantin Apetrei

**Affiliations:** Department of Chemistry, Physics and Environment, Faculty of Sciences and Environment, “Dunărea de Jos” University of Galati, 47 Domnească Street, RO-800008 Galati, Romania; ancuta.dinu@ugal.ro

**Keywords:** biosensor, tyrosine, laccase, polypyrrole, cyclic voltammetry, hexacyanoferrate

## Abstract

Stress, a state of body tension, sometimes caused by increased levels of tyrosine (Tyr) in the body, can lead to serious illnesses such as depression, irritability, anxiety, damage to the thyroid gland, and insomnia. The body can be provided with an adequate concentration of tyrosine by taking pharmaceutical products or by dietary intake. Therefore, this study presents the development of a new enzyme sensor for the quantification of Tyr in pharmaceuticals. A screen-printed carbon electrode (SPCE) was modified with the conductive polymer (CP) polypyrrole (PPy) doped with hexacyanoferrate (II) anion (FeCN), the polymer having been selected for its excellent properties, namely, permeability, conductivity, and stability. The enzyme laccase (Lacc) was subsequently immobilized in the polymer matrix and cross-linked with glutaraldehyde, as this enzyme is a thermostable catalyst, greatly improving the performance of the biosensor. The electrochemical method of analysis of the new device, Lacc/PPy/FeCN/SPCE, was cyclic voltammetry (CV), and chronoamperometry (CA) contributed to the study of changes in the biosensor with doped PPy. CV measurements confirmed that the Lacc/PPy/FeCN/SPCE biosensor is a sensitive and efficient platform for Tyr detection. Thus, this enzyme sensor showed a very low limit of detection (LOD) of 2.29 × 10^−8^ M, a limit of quantification (LOQ) of 7.63 × 10^−8^ M, and a very high sensitivity compared to both devices reported in the literature and the PPy/FeCN/SPCE sensor. Quantitative determination in pharmaceuticals was performed in L-Tyr solution of different concentrations ranging from 0.09 to 7 × 10^−6^ M. Validation of the device was performed by infrared spectrometry (FT-IR) on three pharmaceuticals from different manufacturers and with different Tyr concentrations.

## 1. Introduction

Electrochemical biosensors, designated by scientific researchers as modern analytical devices for the detection of various organic or inorganic compounds, facilitate the performance of many activities in everyday life, activities that require financial efforts, time, and inefficiency [1,2]. Thus, the objective of this paper is to develop an enzyme sensor for the detection of the amino acid (AA) L-Tyr in pharmaceuticals by the cyclic voltammetry (CV) method. The detection of L-Tyr in pharmaceuticals is of great importance for the quality control of pharmaceutical products, in the monitoring of the stability of the pharmaceuticals during storage, and in studies regarding interaction with other active compounds or excipients. Furthermore, knowing the concentration of L-Tyr in a pharmaceutical product can provide accurate information about the bioavailability of the drug in the body and the amount of active substance in relation to the rate at which it is absorbed into the body. The essence of a biosensor is to convert the biological response into an electrical signal, and to manufacture it, materials such as enzymes, antibodies, and nucleic acids are used, which requires multidisciplinary research in chemistry, medicine, pharmacy, biology, and engineering [3,4].

One of the reasons for the present investigation is stress, which is an unavoidable part of human existence and which can take extreme forms, causing numerous mental disorders such as depression, anxiety, schizophrenia, post-traumatic stress disorder, sleep disorders, and thyroid disorders [5,6,7].

**Figure 1:**
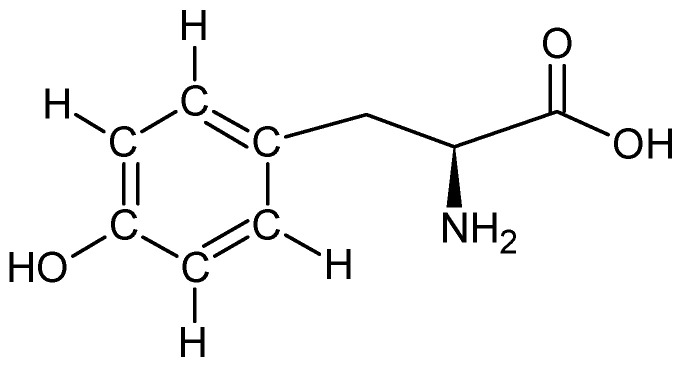
Chemical structure of the L-Tyrosine isomer.

This L-Tyrosine is hydrophobic, non-polar, and has a phenolic hydroxyl group, which gives it a distinctive reactivity, as shown in Figure 1 [8].

It has been scientifically proven that Tyr is beneficial in reducing the damaging physical effects of stress hormones and that it has notable contributions in the treatment of mental disorders, especially in lowering the depression level, on the one hand through the administration of pharmaceutical products containing this AA, and on the other hand through dietary intake of foods rich in Tyr, such as fish, meat, eggs, cheese, etc. [9,10]. For the present study, Tyr was selected to be detected from pharmaceutical products, as it is one of the non-essential AAs of the human body that is used to synthesize proteins, and when insufficient amounts are ingested, the body undergoes a series of changes at the level of the central nervous system [11]. Tyr has two isomers: L-Tyr and D-Tyr, synthesized in vivo from the essential AA L-Phe [12]. The WHO stresses a recommended daily intake of Tyr and Phe of 25 mg/kg body weight [13], showing that an increased level of Tyr can lead to fits of depression [14], Parkinson’s [15], and emotional disorders, and a low level to phenylketonuria (PKU) [16,17], albinism [18], and alkaptonuria [9]. Tyrosinemia is a hereditary, autosomal recessive disease that consists of the incorrect metabolism of Tyr, affecting the liver, kidneys, and peripheral nerves, and for this reason Tyr is measured in all newborn screening programs [19].

In the paper by Dinu and Apetrei, published in 2021, the PPy/FeCN/SPCE sensor was developed for the detection of L-Tyr in pharmaceuticals, obtaining an LOD of 8.2 × 10^−8^ M and exhibiting good electroanalytical performance, i.e., fast response and better sensitivity and stability than the sensors developed in the literature [20]. This study continued this research direction, turning the sensor into a biosensor. Thus, in addition to modifying the sensor with FeCN-doped Ppy-conducting polymer, the enzyme Lacc was immobilized using glutaraldehyde, an enzyme studied since the 19th century [21]. There are several enzymes that can be used for the detection of amino acids (AAs), the most common being tyrosinase, but Lacc was selected for this study, as it is easy to use, requiring no co-factors and participating in many oxidation reactions for a wide variety of organic substances (polyamines, polyphenols, diamines, lignins) [22]. In addition, the presence of the enzyme led to a biosensor with much higher performance compared to the previously obtained sensor, i.e., increased stability, conductivity, reproducibility, and sensitivity; easy preparation; and possibility of use at neutral pH.

Over time, a variety of methods have been used for the detection of Tyr, such as chemiluminescence [23], liquid chromatography–mass spectrometry (LC-MS) [24], fluorimetry [25], UV high-performance liquid chromatography (HPLC) [26], and capillary electrophoresis [27], methods that present a series of limitations: expensive equipment, long analysis time, and possibilities of working in a limited concentration range. In this respect, electrochemical methods such as CV [28,29], differential pulse voltammetry (DPV) [30,31], square wave voltammetry (SWV) [32], and linear sweep voltammetry (LSV) [33,34] have been developed and applied, with several advantages: a wide linear range of biosensor response, cheap equipment, and high stability [35].

In the present study, three methods were used to develop the biosensor: chronoamperometry—useful in the stage of modifying the electrode with doped PPy, cyclic voltammetry to characterize the electrode performance, and the FT-IR method to validate the results obtained.

The novelty of this work is the quantification of tyrosine in pharmaceutical products by means of a device developed in the laboratory, using a conductive polymer doped with an anionic agent and an enzyme fixed with a cross-linking agent on a screen-printed carbon electrode, which, following electrochemical analysis by the cyclic voltammetry method, obtained increased performance, sensitivity, and selectivity. Currently, there are no studies on tyrosine detection with a PPy- and Lacc-modified enzyme sensor, and future research directions should consider the application of the biosensor on biological fluids.

## 2. Materials and Methods

### 2.1. Chemicals and Reagents

Screen-printed carbon electrodes (SPCE), model DRP-C110, diameter of 4 mm, surface of 12.56 mm^2^, were purchased from Metrohm DropSens (Oviedo, Spain). The following chemicals were purchased from Sigma-Aldrich (St. Louis, MO, USA): pyrrole (98%), FeCN (≥99.5%), laccase (Lacc), glutaraldehyde (GA), L-Tyr (≥98%), and potassium chloride (≥99.0%) (KCl). The real samples were represented by products purchased from the pharmaceutical market. Ultrapure water (18.3 MΩ·cm, Milli-Q Simplicity^®^ Water Purification System from Millipore Corporation—Millipore, Bedford, MA, USA) was used to prepare solutions of these compounds.

### 2.2. Instruments and Methods

The EG&G potentiostat/galvanostat (Princeton Applied Research, Oak Ridge, TN, USA) 263A model, controlled by ECHEM software, was the device used to modify the working electrode with FeCN-doped PPy by the CA method. Three electrodes were connected to this device: the Ag/AgCl/KCl_3M_ reference electrode, the platinum (Pt) wire auxiliary electrode, and the SPCE working electrode, simultaneously introduced into the electrochemical cell.

CV, the method used for biosensor characterization, was applied using the Biologic SP 150 potentiostat/galvanostat (Bio-Logic Science Instruments SAS, Seyssinet-Pariset, France) controlled by EC-Lab Express software. In this case, a 15 mL electrochemical cell was used, in which the three electrodes were immersed: the Lacc/Ppy/FeCN/SPCE working electrode, the reference electrode, and the auxiliary electrode (counter electrode–carbon, Ag/AgCl reference electrode). The working parameters of CV were different scan rates of 0.1–1.0 V·s^−1^ and the optimal potential range was between −1.0 and 0.5 V. The analyzed solutions used for the electrochemical studies were 0.1 M KCl solution and a double solution of 0.1 M KCl-10^−3^ M L-Tyr. The voltammograms obtained were analyzed using two programs: ORIGIN vs. 6.0 Professional and Microsoft Excel.

For biosensor validation, the FT-IR method was applied to the Bruker ALPHA FT-IR spectrophotometer (BrukerOptik GmbH, Ettlingen, Germany) in the range of 4000–500 cm^−1^, controlled by OPUS software (BrukerOptik GmbH, Ettlingen, Germany).

The Elmasonic S10H device (ultrasonic bath) was used for homogenization and dissolution of solutions.

### 2.3. Process for the Preparation of the PPy/FeCN/Lacc/SPCE Enzyme Sensor

#### 2.3.1. Preparation of the Monomer/Doping Agent Solution (PPy/FeCN)

A solution of exact concentration obtained from pyrrole, potassium hexacyanoferrate (II) (FeCN), and KCl of 0.1 M pyrrole/0.1 M FeCN/0.1 M KCl was used to modify the sensor. To homogenize the three compounds, the flask containing the prepared solution was placed in the ultrasonic bath apparatus for 5 min. The deposition was achieved by connecting the DRP-C110 sensor to the electrochemical cell and introducing the three electrodes into the monomer/doping-agent solution, applying a potential of 0.8 V for 90 s at a constant temperature of 25 °C. Subsequently, these prepared sensors were rinsed with ultrapure water. The method to characterize the changes occurring on the sensor working surface was chronoamperometry.

#### 2.3.2. Manufacture of the PPy/FeCN/Lacc/SPCE Biosensor

**Figure 2:**
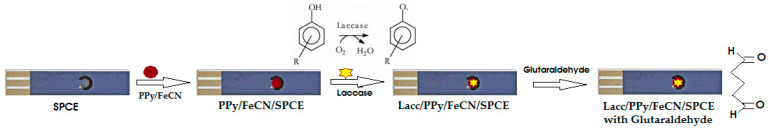
Schematic diagram of the immobilization of the Lacc enzyme in the construction of the biosensor.

The transformation of the PPy/FeCN/SPCE sensor into the PPy/FeCN/Lacc/SPCE biosensor was achieved by droplet pouring the Lacc enzyme onto the working surface of the electrode, equivalent to 10 μL enzyme. This stage was followed by cross-linking with glutaraldehyde (GA) reagent and drying. The biosensor preparation technique is also called the drop-and-dry technique. To obtain the laccase solution, 0.05 g laccase dissolved in 0.1 M KCl solution was used, of which 2 μL was added in five steps to the working surface of the PPy/FeCN-modified electrode and placed on top of 2% glutaraldehyde vapor for 1 min, a procedure called cross-linking, illustrated in Figure 2. Many researchers have used GA prior to enzyme immobilization because cross-linking decreases the rate of enzyme deactivation, but few have attempted the cross-linking process after enzyme application [36]. Before using the biosensor, it was kept for 5–10 min at room temperature. The prepared biosensors were stored at 4 °C in darkness until use.

The characterization of these biosensors was performed by electrochemical measurements, applying the CV method, with the following pre-set parameters: initial potential 0.0 V, positive vertex potential 0.5 V, negative vertex potential −1.0 V, and a scan rate between 0.1 and 1.0 V·s^−1^.

### 2.4. Analysis of Real Samples

Three products purchased from the pharmaceutical market from different manufacturers were analyzed. The criteria for the selection of these products were the Tyr content and the different concentrations of Tyr in the pharmaceutical products. Cebrium, manufactured by EVER NEURO PHARMA GmbH Austria, is a dietary supplement with a unique combination of amino acids specific to the nervous system, effectively supporting mental abilities, indicated in conditions of stress, overwork, and mild forms of anxiety and depression. Each capsule contains 68 mg of amino acids, where Tyr is found in a concentration of 4.012 mg per capsule.

The second product, Tiroidin, comes from the manufacturer PARAPHARM Romania and, besides the 90 mg Tyr, contains spirulina, vitamin E, selenium, and iodine. The last product, L-Tyrosine SOLARAY U.S., with a content of 500 mg L-Tyr obtained by bacterial fermentation, is intended for people who suffer from fatigue, depression, or excessive sleepiness, or in situations where their body cannot synthesize the necessary phenylalanine. On the other hand, it increases focus and learning capacity, attention, and motivation, and improves the body’s ability to cope with stress factors.

## 3. Results

Since the sensor-doping process by the CA method was mentioned in the paper by the same authors in which the PPy/FeCN/SPCE sensor was fabricated [20], this section will highlight the importance of the Lacc enzyme in the detection of Tyr in pharmaceuticals, mentioning the performance of the new biosensor compared to the sensor.

### 3.1. Cyclic Voltammetry Characterization

The CV method was used to characterize the SPCE electrode modified with monomer/doping-agent solution and laccase. Thus, the three electrodes, i.e., the working electrode, the counter electrode, and the reference electrode, were immersed in two solutions: an inactive solution of 0.1 M KCl and a double solution of 0.1 M KCl-10^−3^ M L-Tyr, recording voltammograms in the potential range of −1.0 to 0.5 V.

#### 3.1.1. Stable Electrochemical Responses of Biosensors in 0.1 M KCl Solutions and in a Double Solution of 0.1 M KCl-10^−3^ M L-Tyr

**Figure 3:**
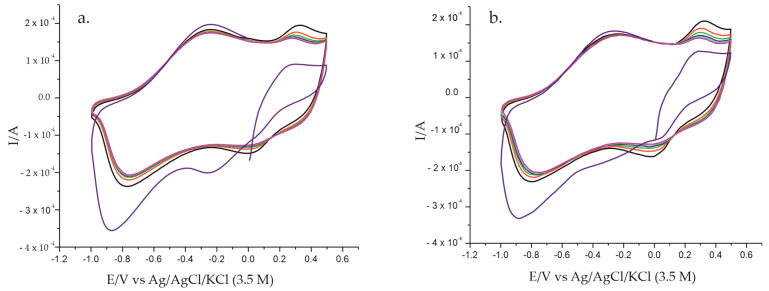
Stabilization of the Ppy/FeCN/Lacc/SPCE biosensor response immersed in (**a**) 0.1 M KCl solution; (**b**) 0.1 M KCl-10^−3^ M L-Tyr double solution.

After doping PPy with FeCN by the CA method and after the immobilization of the Lacc enzyme on the electrode surface, the biosensor was placed in a 0.1 M KCl solution. Initially, the behavior of the biosensor in KCl solution was analyzed, as it is an inactive solution that allows the subsequent detection of specific Tyr peaks. For the stability of the biosensor, six cycles were recorded at a scan rate of 0.1 V·s^−1^, during which time PPy and Lacc stabilization in the electrolyte solution took place. This is illustrated in Figure 3a.

Furthermore, for another biosensor prepared under the same conditions, six cycles were recorded for stabilization purposes at the same scan rate but in double solution, i.e., in 0.1 M KCl-10^−3^ M L-Tyr (Figure 3b). The stable responses in electrolyte solutions were recorded starting with the sixth cycle in both cases, the first cycles having a different representation compared to the subsequent ones. Increased anodic and cathodic peak intensities were observed for the sensor immersed in double solution. Thus, in Table 1 the peak intensities for both redox systems at the same potential for the sixth cycle after stabilization of the PPy/FeCN/Lacc/SPCE biosensor immersed in the two solutions are reported.

**Figure 4:**
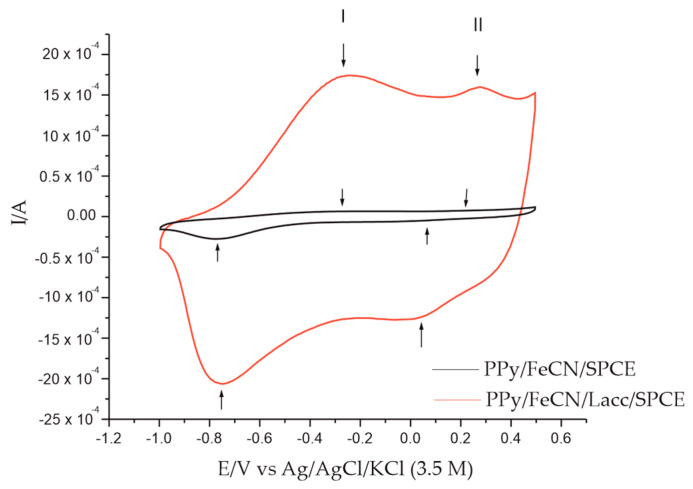
Stable responses of the PPy/FeCN/SPCE sensor and the PPy/FeCN/Lacc/SPCE biosensor immersed in 0.1 M KCl.

A comparative study was also carried out on the stable responses of the non-enzymatic sensor with those of the enzymatic sensor, first in inactive 0.1 M KCl solution at the same scan rate, i.e., 0.1 V·s^−1^. The recorded voltammetric response is illustrated in Figure 4 and accompanied by Table 2, which details the intensities and potential of the peaks obtained with both devices.

As shown in Figure 4 and Table 2, the benefits of the immobilized enzyme on the Ppy/FeCN/SPCE sensor can easily be observed. In the case of the biosensor, the I_pc_/I_pa_ ratio showed the value closest to the ideal value, i.e., 1, especially in the case of redox system I, the system corresponding to the redox processes of PPy. The peak intensities of the biosensor had increased values, especially in the cathodic scan, compared to those of the sensor, due on the one hand to the conductive polymer and the anion, and on the other to the Lacc enzyme, which conferred an increased sensitivity to the biosensor. A reduction in the background current for the biosensor can also be observed, with the differences between the two devices being obvious.

**Figure 5:**
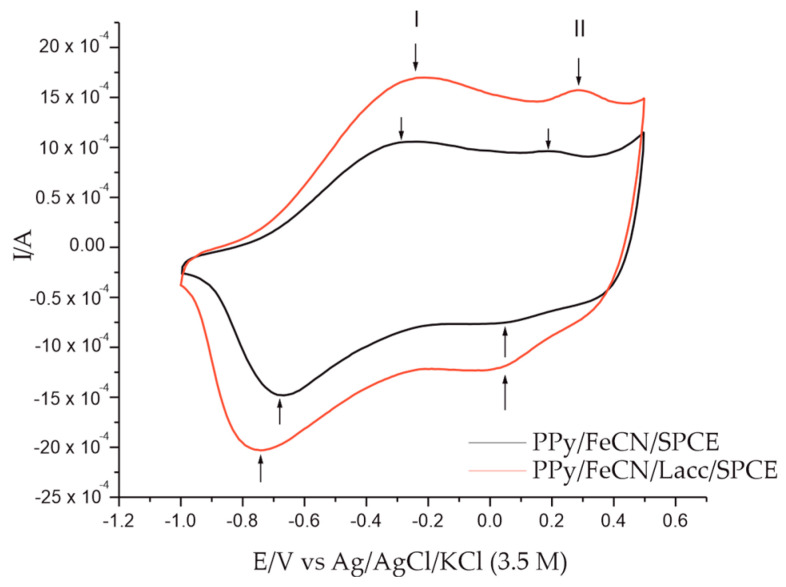
Stable response of the PPy/FeCN/SPCE sensor and the PPy/FeCN/Lacc/SPCE biosensor immersed in 0.1 M KCl–10^−3^ M L-Tyr double solution. I—Redox system I; II—Redox system II.

The next test of the biosensor was to immerse it in the double solution of 0.1 M KCl and 0.1 M KCl-10^−3^ M L-Tyr under the same conditions and parameters used for the inactive solution. The stable response is illustrated in Figure 5. The values of the electrochemical parameters obtained in this step are listed in Table 3.

A first noteworthy observation is the difference obtained by the non-zero electrode in the two solutions, 0.1 M KCl and the double solution 0.1 M KCl-10^−3^ M L-Tyr. This is explained by the presence of more intense peaks in the double solution due to the presence of AA L-Tyr. Another significant difference is that on the one hand, between the sensor and the biosensor, the intensities of the peaks were increased both at the level of redox system I, corresponding to PPy, and at the level of redox system II, corresponding to the potassium ferrocyanide included in the polymer matrix, and on the other hand the Lacc enzyme provided better selectivity and accuracy to the biosensor.

#### 3.1.2. Effect of the Scan Rate on PPy/FeCN/Lacc/SPCE Immersed in Double Solution 0.1 M KCl and 0.1 M KCl-10^−3^ M L-Tyr

**Figure 6:**
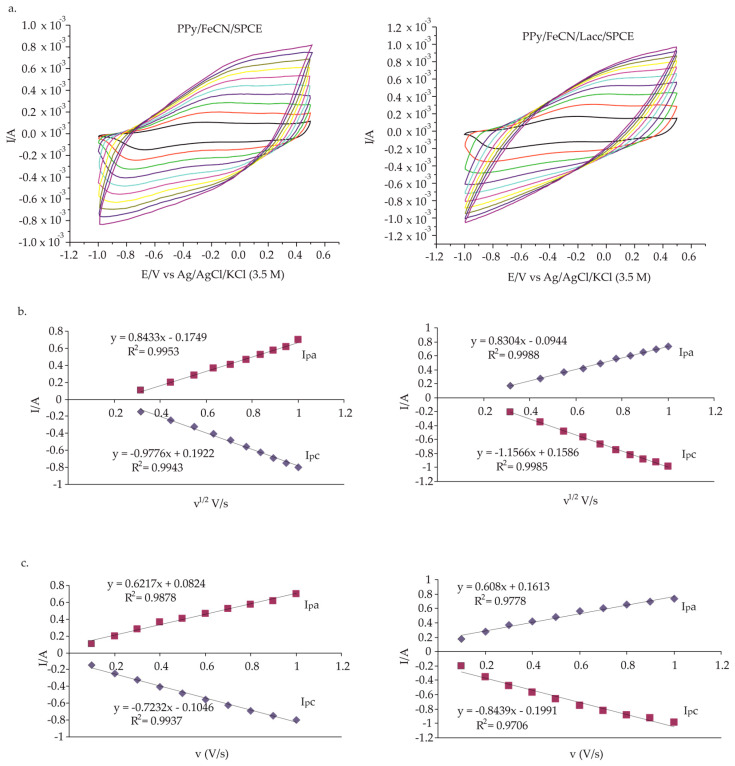
(**a**) CVs for PPy/FeCN/SPCE at different scan rates of 0.1–1.0 V·s^−1^ in the double solution of 0.1 M KCl and 0.1 M KCl-10^−3^ M L-Tyr; (**b**) dependence between the square root of the scan rates and the intensity of the anodic and cathodic peaks; (**c**) dependence between scan rate and intensity of the anodic and cathodic peaks.

At this stage, the influence of the scan rate was studied for the PPy/FeCN/Lacc/SPCE biosensor immersed in 0.1 M KCl-10^−3^ M L-Tyr electrolyte solution, with cyclic voltammograms recorded at different scan rates, i.e., between 0.1 and 1.0 V·s^−1^, using the same potential range mentioned in the previous stage. The kinetic parameters can be investigated with the Tafel plot [37], illustrated in Figure 6, when the mechanism is diffusion. The oxidation and reduction peaks were in close dependence with the scan rate, and increasing the latter led to higher peak intensities (Figure 6a). Figure 6b shows the linear dependence between the peak or peak current intensity and the square root of the scan rate, with the correlation coefficient being 0.9988 for the anodic peak and 0.9985 for the cathodic peak.

The curve obtained for the cathodic peak of redox system I for both the biosensor and non-enzymatic sensor, extracted from the intensity plot as a function of the square root of the scan rate, was thus considered. Subsequently, the active area for both electrodes was calculated, and the values obtained are given in Table 4.

Under these circumstances, according to the data obtained, the redox process was influenced by the diffusion process. The diffusion coefficient for L-Tyr is 3 × 10^−5^ cm^2^·s^−1^, a value that allowed for the calculation of the active area of the PPy/FeCN/Lacc/SPCE biosensor, as shown in Table 4. The Randles–Sevcik equation describes the influence of the scan rate against peak intensity. According to this equation, the cathodic peak intensity (I_pc_, A) is the product of 268.600, the number of electrons transferred in redox processes (n^3/2^), the area of the electrode active surface (A/cm^2^), the diffusion coefficient (D, cm^2^·s^−1^), the concentration (C, mol·cm^−3^), and the scan rate (v^1/2^, V·s^−1^) [38].

Therefore, according to the graphs in Figure 4, Figure 5 and Figure 6, it can be seen that, in addition to the electropolymerization of pyrrole and the general doping process [39], the action of the metalloenzyme Lacc, which is an oxidoreductase and in which the electron transfer is via copper ions, was also involved. Thus, Lacc catalyzed the oxidation reaction of Tyr to 3,4-dioxyphenylalanine, where it oxidized to the respective quinone, which appeared as an intermediate in the synthesis of melanin pigments [40]. The mechanism of the enzymic action is illustrated in the following reactions:

Tyr + Lac (oxy) → 3,4- dioxyphenylalanine + Lac (deoxy) + 2H^+^ + 2e^−^ (1)

Lac (deoxy) + O_2_ + 4H^+^ → Lac (oxy) + 2H_2_O (2)

The redox process at the biosensor surface showed a quasi-reversible nature, with the anodic peak potential shifting to some extent towards the positive potential as the scanning rate increased, whereas the cathodic peak potential shifted moderately towards the negative potential.

#### 3.1.3. Calibration Curve and Detection Limit of the PPy/FeCN/Lacc/SPCE Biosensor

**Figure 7:**
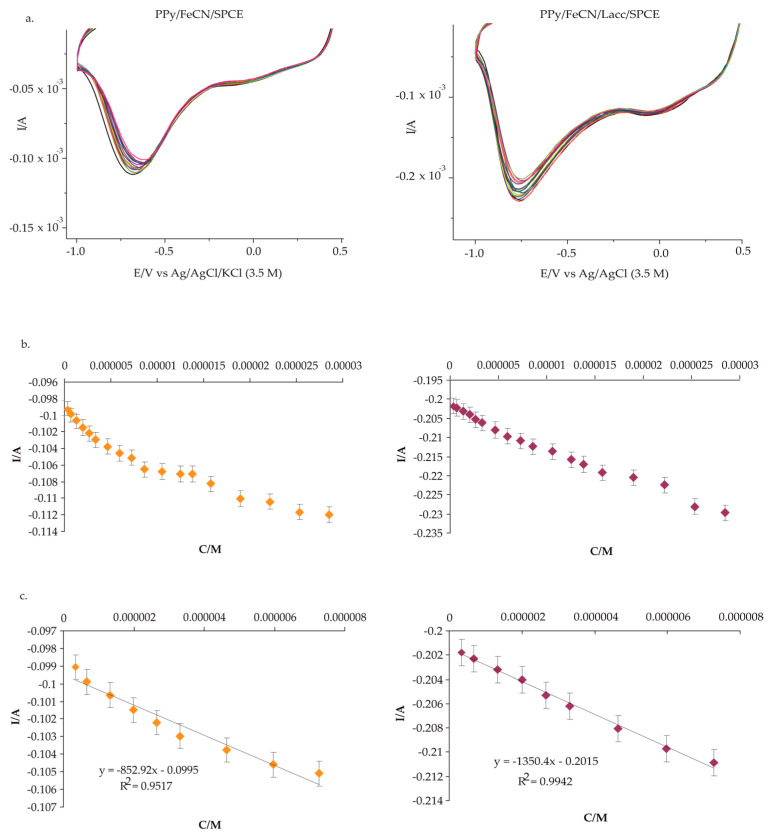
(**a**) The relationship between the cathodic peak of the sensor and the biosensor, and the concentration of the 0.1 M KCl-10^−3^ M L-Tyr solution; (**b**) the cathodic peak current I variation with Tyr concentration; (**c**) the sensor and biosensor calibration curves in the concentration range of 0.09–7 × 10^−6^ M and 0.2–7 × 10^−6^ M, respectively.

Cyclic voltammograms for the detection of L-Tyr in solutions of different concentrations of 0.1 M KCl-10^−3^ M L-Tyr, in the potential range of −1.0 to 0.5 V and in the concentration range of 0.09–27 × 10^−6^ M, were obtained for the two devices, as shown in Figure 7.

Figure 7a displays the relationship between the cathodic current of the sensor and the biosensor, and the concentration of L-Tyr by the CV technique. The linearity ranges identified in Figure 7b were between 0.09 and 7 × 10^−6^ M for the sensor and from 0.2 to 7 × 10^−6^ M for the biosensor. Figure 7c illustrates the calibration curves for both the sensor and the biosensor, with correlation coefficients R^2^ equal to 0.95 for the sensor and 0.99 for the biosensor, and a detection limit of 3.76 × 10^−7^ for the sensor and 2.29 × 10^−8^ for the biosensor (Table 5).

#### 3.1.4. Quantitative Determination of L-Tyr at PPy/FeCN/Lacc/SPCE in Pharmaceutical Samples by the CV Method and Standard FT-IR Method for Biosensor Validation

The selection of samples for biosensor validation was carried out for the purpose of quality control of the pharmaceutical products, as there is no research to date with an enzyme sensor for such a study. Because there are many products with different concentrations of tyrosine from different manufacturers on the pharmaceutical market, only three pharmaceutical products were selected and investigated similarly by CV for this study: Cebrium, with 4.012 mg per capsule of L-Tyr; Tiroidin, with 90 mg per capsule of L-Tyr; and L-Tyrosine, with 500 mg per capsule of L-Tyr.

The analytical performance of the PPy/FeCN/Lacc/SPCE biosensor demonstrated selective detection of Tyr, including from multicomponent solutions, such as the solution obtained from the Cebrium product. The Lacc enzyme gave the biosensor increased efficiency and accuracy over this AA.

**Figure 8:**
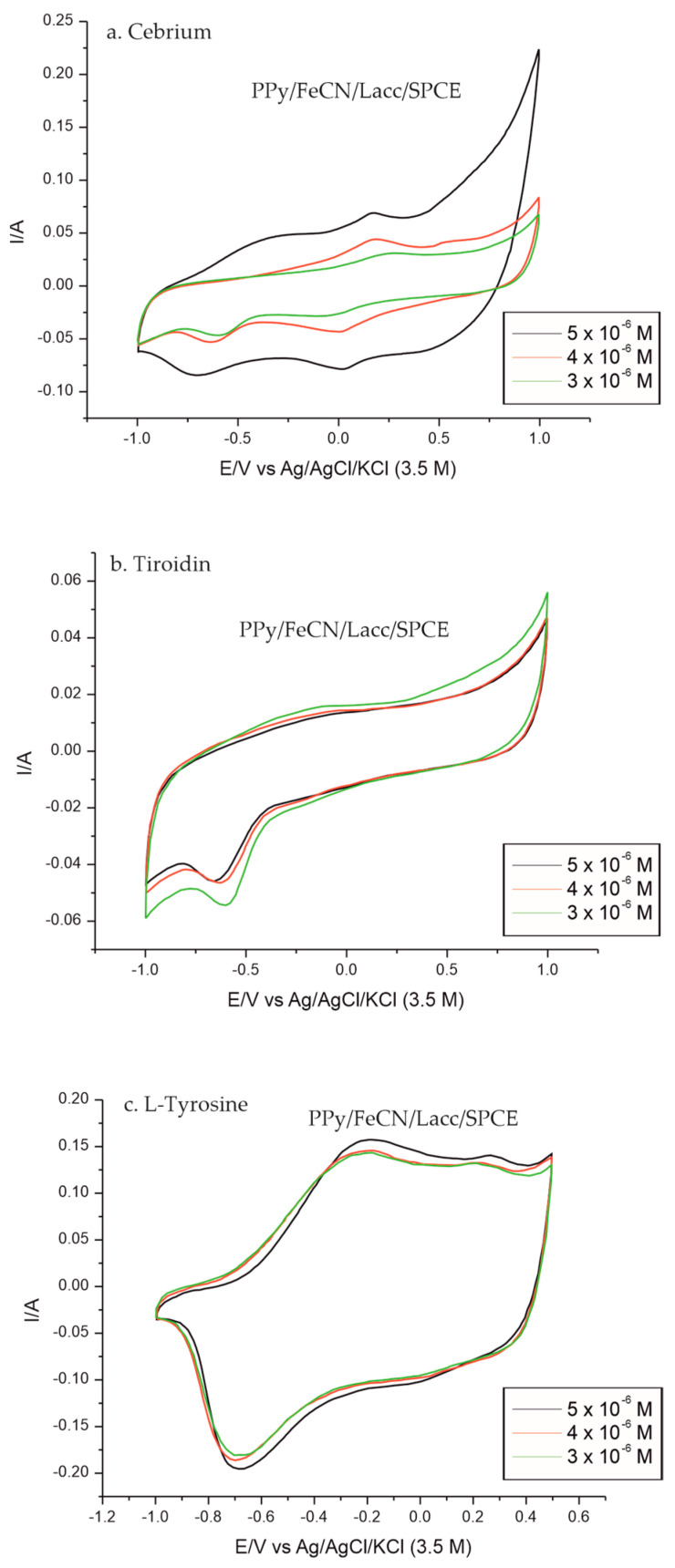
Cyclic voltamograms of the PPy/FeCN/Lacc/SPCE biosensor, recorded at a scan rate of 0.1 V·s^−1^ at three concentrations of 3 × 10^−6^, 4 × 10^−6^, and 5 × 10^−6^ M and immersed in (**a**) Cebrium solution, (**b**) Tiroidin solution, and (**c**) L-Tyrosine solution.

Quantitative determination of L-Tyr was performed by the standard addition method for each pharmaceutical product. Figure 8 shows the CV responses of the PPy/FeCN/Lacc/SPCE biosensor prepared for each sample. The prepared solutions of each pharmaceutical product had different concentrations of L-Tyr: 3 × 10^−6^, 4 × 10^−6^, and 5 × 10^−6^ M.

Biosensor validation was performed by the FT-IR method. The electroanalytical results for the quantification of L-Tyr obtained by the CV method based on the biosensor developed in this study were compared both with those obtained by the FT-IR method and with those provided by the producers of the pharmaceutical products under analysis. The results obtained are included in Table 6.

Measurements with the biosensors prepared under the same conditions led to the following coefficient of variation results (Table 6).

The quantification of L-Tyr by the FT-IR method was performed by analyzing three standards obtained by mixing pure L-Tyr with KBr, with concentrations similar to those of the studied pharmaceuticals: 5 mg/g, 100 mg/g, and 500 mg/g. For the quantitative determination of L-Tyr, the absorbance of the peak at 1650 cm^−1^, characteristic of the N−H group vibration (bending), was taken into account.

As can be observed in Table 6, the L-Tyr concentration values of the results obtained by the CV method and the FT-IR method, and those provided by the pharmaceutical manufacturers, are similar, with small differences, which demonstrates the accuracy of the L-Tyr quantification method developed in this biosensor-based research. Therefore, the biosensor could be applied in laboratory practice in the quality control of pharmaceutical products containing L-Tyr, but also for analyses of L-Tyr in biological fluids.

Finally, measurements were made in real samples with the biosensor using the standard addition method. In order to measure real samples, three capsules of Tiroidin were crushed and homogenized, and after this, some of the powder was dissolved in 0.1 M KCl solution to prepare a stock solution. Insoluble excipients were separated by filtration. Using the stock solution, a 1.5 μM L-Tyr solution was prepared by dilution and the biosensor response in this solution was recorded. Then, 1.5 μM L-Tyr was added to the real sample solution and the biosensor response was recorded again. As shown in Table 7, the analytical recoveries were good, and the relative standard deviation for seven successive determinations was less than 5%. This study shows that the biosensor showed good analytical performance in real samples.

These results highlight the applicability of the biosensor in the pharma-medical field. The recovery percentage clearly demonstrates the efficiency of the voltammetric method in the analysis and quantification of L-Tyr in pharmaceuticals.

### 3.2. Repeatability, Reproducibility, Stability, and Interference Study of the Biosensor

Series of five successive CV measurements with 10^−5^ M Tyr, each recorded on a new modified biosensor, produced relative standard deviations of 2.8%. The results indicate that the biosensor provides good reproducibility in the electrochemical detection of L-Tyr.

The stability of the biosensor in the detection of L-Tyr was also determined when the biosensor was stored in the refrigerator for 15 days. The response was recorded in 10^−5^ M solution, and L-Tyr was found to be stable, maintaining 90% of its original intensity.

**Figure 9:**
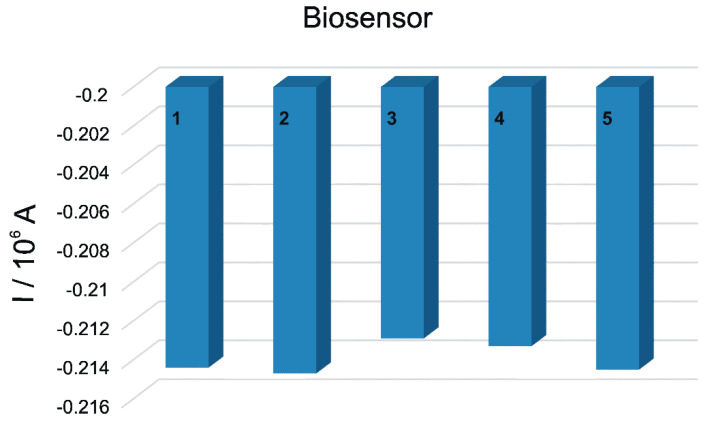
Stability of the responses for 10^−5^ M L-Tyr obtained with five different modified PPy/FeCN/Lacc/SPCE biosensors (RSD = 0.33%).

The reproducibility of the fabrication process was also studied. Five biosensors were prepared in identical conditions and the responses in 10^−5^ M L-Tyr were registered. As can be observed in Figure 9, the differences between the biosensor responses were small, with the relative standard deviation (RSD) being 2.7%.

The substances used in the interference studies were a series of amino acids with similar chemical structure to Tyr, namely, Phe, tryptophan (Trypt), and cysteine. These amino acids were dissolved in a 0.1 M KCl solution.

Under the experimental conditions described above, the effects of a series of AAs with similar chemical structure to Tyr, Phe, Trypt, and cysteine were evaluated. Phe, Trypt, and cysteine had low influence on the biosensor response in 0.1 M KCl solution.

Experimental results (Table 8) show that both substances had almost no interference with the determination of L-Tyr. Therefore, it can be concluded that the proposed method is capable of analyzing L-Tyr in the presence of interfering substances and consequently can be considered specific. The average signal change was found to be 0.42% for the peak potential and 1.64% for the current.

## 4. Discussion

Comparing the values obtained by the two devices in terms of both linearity range and detection limit, it is evident that the enzyme sensor showed increased performance, thus demonstrating the importance of the enzyme imprinted on the surface of the working electrode, which makes it more sensitive, as well as the favorable interaction of the Lacc enzyme with tyrosine.

Table 9 shows the performance of some biosensors used for the detection of Tyr by electrochemical methods, the enzymes used for electrode modification, and the samples on which the enzyme sensors were tested. The use of the Lacc enzyme was identified in only one scientific study, thus highlighting the necessity and effectiveness of the new device developed, but no study on the detection of tyrosine in pharmaceutical products has been conducted so far, which means our new device finds its usefulness and applicability in the quality control of such products.

The biosensor proposed in this study demonstrated increased sensitivity and acceptable performance due to modification with FeCN-doped PPy, also indicating that immobilization of the Lacc enzyme could help to increase the performance of the biosensor for L-Tyr detection.

## 5. Conclusions

In this study, a new biosensor was developed by immobilizing the laccase enzyme on the surface of an electrode modified with a conductive material with excellent properties, PPy polymer doped with FeCN anion, by chronoamperometry. Characterization of the modified electrode was performed by voltammetric techniques and FT-IR spectroscopy, and the results obtained demonstrated the increased selectivity of the biosensor for the quantitative determination of Tyr compared to those obtained by the unmodified electrode with the Lacc enzyme. In addition, Lacc and CP demonstrated biocompatibility, superior mechanical properties, and a high surface-to-volume ratio for the biosensor. The concentration range in which PPy/FeCN/Lacc/SPCE was tested was in the range of 0.2–6 × 10^−6^ M and the detection limit obtained was 2.29 × 10^−8^ M, a low value compared to the non-enzymatic sensor and other devices reported in the literature. Moreover, this new biosensor demonstrated good stability for one week and acceptable recoveries when tested on real samples, i.e., pharmaceuticals with different Tyr concentrations. The development of this biosensor can prove effective in controlling the quality of pharmaceutical products containing L-Tyr and is a challenge for future research, in the sense of developing a biosensor to detect the level of L-Tyr in food and biological fluids from birth, as such a device can help prevent many diseases.

## Figures and Tables

**Table 1:** Electrochemical parameters of PPy/FeCN/Lacc/SPCE biosensor immersed in two electrolyte solutions: 0.1 M KCl and 0.1 M KCl-10^−3^ M L-Tyr.

Biosensor Electrolyte Solution		Redox System I				Redox System II			
		E_pa_ ^1^ (V)	I_pa_ ^2^ (A)	E_pc_ ^3^ (V)	I_pc_ ^4^ (A)	E_pa_ (V)	I_pa_ (A)	E_pc_ (V)	I_pc_ (A)
PPy/FeCN/Lacc/SPCE	0.1 M KCl	−0.23	1.6 × 10^−4^	−0.73	−2.05 × 10^−4^	0.29	1.5 × 10^−4^	0.04	−1.23 × 10^−4^
	0.1 M KCl–10^−3^ M L-Tyr		1.7 × 10^−4^		−2.06 × 10^−4^		1.6 × 10^−4^		−1.24 × 10^−4^

^1^ E_pa_, anodic peak potential; ^2^ I_pa_, anodic peak current; ^3^ E_pc_, cathodic peak potential; ^4^ I_pc_, cathodic peak current.

**Table 2:** Electrochemical parameters obtained from the voltamograms of the PPy/FeCN/SPCE sensor and the PPy/FeCN/Lacc/SPCE biosensor immersed in 0.1 M KCl solution.

Electrode		Electrochemical Parameters					
		E_pa_ ^1^ (V)	E_pc_ ^2^ (V)	ΔE ^3^ (V)	I_pa_ ^4^ (A)	I_pc_ ^5^ (A)	I_pc_/I_pa_
PPy/FeCN/SPCE sensor [20]	Redox system I	−0.32	−0.74	0.42	6 × 10^−6^	−2 × 10^−5^	4.31
	Redox system II	0.20	0.03	0.17	7.2 × 10^−6^	−4.9 × 10^−6^	0.69
PPy/FeCN/Lacc/SPCE biosensor	Redox system I	−0.26	−0.74	0.48	1.7 × 10^−4^	−2 × 10^−4^	1.18
	Redox system II	0.26	0.03	0.23	1.5 × 10^−4^	−1.2 × 10^−4^	0.78

^1^ E_pa_, anodic peak potential; ^2^ I_pa_, anodic peak current; ^3^ ΔE = E_pa_-E_pc_; ^4^ E_pc_, cathodic peak potential; ^5^ I_pc_, cathodic peak current.

**Table 3:** Electrochemical parameters obtained from the voltamograms of the PPy/FeCN/SPCE sensor and the PPy/FeCN/Lacc/SPCE biosensor immersed in 0.1 M KC-10^−3^ M L-Tyr solution.

Electrode		Electrochemical Parameters					
		E_pa_ ^1^ (V)	E_pc_ ^2^ (V)	ΔE ^3^ (V)	I_pa_ ^4^ (A)	I_pc_ ^5^ (A)	I_pc_/I_pa_
PPy/FeCN/SPCE sensor [20]	Redox system I	−0.28	−0.66	−0.38	1.2 × 10^−4^	−1.3 × 10^−4^	1.09
	Redox system II	0.19	0.08	0.11	9.4 × 10^−5^	−8.5 × 10^−5^	0.90
PPy/FeCN/Lacc/SPCE biosensor	Redox system I	−0.20	−0.74	0.54	1.7 × 10^−4^	−2 × 10^−4^	1.19
	Redox system II	0.26	0.04	0.22	1.5 × 10^−4^	−1.1 × 10^−4^	0.75

^1^ E_pa_, anodic peak potential; ^2^ I_pa_, anodic peak current; ^3^ ΔE = E_pa_-E_pc_; ^4^ E_pc_, cathodic peak potential; ^5^ I_pc_, cathodic peak current.

**Table 4:** The area of the active surface of the sensor and the biosensor corresponding to the cathodic peak of redox system I.

Electrode	Solution	Slope	R^2^	Active Area (cm^2^)	Geometric Area	Roughness Factor
PPy/FeCN/SPCE sensor [20]	0.1 M KCl and 0.1 M KCl–10^−3^ M L-Tyr	−0.0009776	0.9943	1.3488	0.1256	10.74
PPy/FeCN/Lacc/SPCE biosensor		−0.0011566	0.9985	1.5957		12.70

**Table 5:** Data obtained for the calibration curves of the Ppy/FeCN/SPCE sensor and the Ppy/FeCN/Lacc/SPCE biosensor at L-Tyr detection.

Electrode	LOD ^1^ (M)	LOQ ^2^ (M)
PPy/FeCN/SPCE sensor [20]	3.76 × 10^−7^	1.25 × 10^−6^
PPy/FeCN/Lacc/SPCE biosensor	2.29 × 10^−8^	7.63 × 10^−8^

^1^ Limit of detection; ^2^ limit of quantification.

**Table 6:** Results obtained by the prepared biosensor regarding the concentration of L-Tyr from the selected pharmaceutical products compared to those mentioned by the manufacturer and determined by the FT-IR method.

Pharmaceutical Product	Manufacturer	L-Tyr in the Pharmaceutical Product/mg per Capsule	L-Tyr Determined by CV/mg per Capsule	L-Tyr Determined by FT-IR/mg per Capsule
Cebrium	EVER Neuro Pharma	4.012	4.124	4.131
Tiroidin	Parapharm	90	92.34	92.98
L-Tyrosine	Solaray	500	477.5	478.3

**Table 7:** Tyr analysis data from the pharmaceutical product Tiroidin.

Sample	Added (μM)	Found (μM)	Recovery (%)
1	1.5	1.46	97.34
2	3.0	3.07	102.34
3	4.5	4.48	99.55
4	6.0	6.85	97.50

**Table 8:** Interference studies on the voltammetric response of 10^−5^ M L-Tyr at PPy/FeCN/Lacc/SPCE.

L-Tyr (10^−5^ M) + Interfering Species (10^−5^ M)	Observed Potential (V)	Potential Change (%)	Average Potential Change (%)	Observed Current (10^6^ A)	Current Change (%)	Average Current Change (%)
L-Tyr	−0.7444	−	0.42	−0.2144	−	1.64
L-Tyr + Phe	−0.7419	0.33		−0.2119	1.66	
L-Tyr + Trypt	−0.7403	0.55		−0.2103	1.91	
L-Tyr + Cysteine	−0.7415	0.38		−0.2115	1.35	

**Table 9:** Detection method, linearity range, and limit of detection of the biosensors developed for Tyr detection.

Nr. Crt.	Electrode Material	Enzyme	Real Samples	Detection Method	Linear Range (M)	LOD (M)	Ref.
1	Tyrosine hydroxylase onto palladium–platinum bimetallic alloy nanoparticles/chitosan-1-ethyl-3-methylimidazoliurn bis(trifluoromethylsulfonyl) imide/graphene-multiwalled carbon nanotubes-IL/glassy carbon electrode (TyrH/PdPt NPs/Ch-IL/Gr-MWCNTsIL/GCE)	Tyrosine hydroxylase	Food	CV, DPV	0.01 × 10^−9^–8.0 × 10^−9^ and 8.0 × 10^−9^–160.0 × 10^−9^	0.009 × 10^−9^	[41]
2	Banana peel tissue tyrosinase/3-mercaptopropyl trimethoxysilane-functionalized silica nanoparticle (B.P.Tyr/M/SN-MPT)	Tyrosinase	Banana peel tissue (*Musa Cavendish*)	CV, DPV	5 × 10^−8^–6 × 10^−4^	2 × 10^−8^	[42]
3	Hemin-modified graphene nanosheet electrode (HGN/GCE)	Hemin	Tyrosine	CV	5 × 10^−7^–2 × 10^−5^	7.5 × 10^−8^	[43]
4	Glassy carbon electrode with tyrosine hydroxylase and reduced graphene oxide (rGO/TyrHAS/GCE)	Tyrosine hydroxylase	Food	CV	1 × 10^−12^–3.45 × 10^−7^	7 × 10^−11^	[44]
5	Tyrosinase enzyme/multi-walled carbon nanotubes/polysulfone/glassy carbon electrode (TyOx/MWCNT/PSF/GCE)	Tyrosinase	Tyrosine	CV	1.96 × 10^−6^–3.94 × 10^−4^	3 × 10^−10^	[45]
6	Organic electrochemical transistor (OECT) by functionalizing a single cotton yarn with semiconducting PEDOT-modified cotton fiber	Fungal laccase POXA1b	Textile fiber	UV-VIS, CV	10^−8^ and 10^−2^	10^−8^	[46]

## Data Availability

The authors confirm that the data supporting the findings of this study are available within the article.
